# Prevention of cardiovascular disease guided by total risk estimations − challenges and opportunities for practical implementation: highlights of a CardioVascular Clinical Trialists (CVCT) Workshop of the ESC Working Group on CardioVascular Pharmacology and Drug Therapy

**DOI:** 10.1177/1741826711424873

**Published:** 2012-12

**Authors:** Faiez Zannad, Jean Dallongeville, Robert J Macfadyen, Luis M Ruilope, Lars Wilhelmsen, Guy De Backer, Ian Graham, Matthias Lorenz, Giuseppe Mancia, David A Morrow, Željko Reiner, Wolfgang Koenig

**Affiliations:** 1 Institut Lorrain du Coeur et des Vaisseaux, Vandoeuvre, France.; 2 Pasteur Institute, Lille, France.; 3 City Hospital, Birmingham, UK.; 4 12 de Octubre Hospital, Madrid, Spain.; 5 University of Gothenburg, Gothenburg, Sweden.; 6 Ghent University, Ghent, Belgium.; 7 Adelaide and Meath Hospital, Dublin, Ireland.; 8 Frankfurt University, Frankfurt, Germany.; 9 University of Milan-Bicocca, Milan, Italy.; 10 Brigham and Women's Hospital, Boston, USA.; 11 University of Zagreb, Zagreb, Croatia.; 12 University of Ulm Medical School, Ulm, Germany.

**Keywords:** Cardiovascular disease, primary prevention, risk-guided therapy

## Abstract

This paper presents a summary of the potential practical and economic barriers to implementation of primary prevention of cardiovascular disease guided by total cardiovascular risk estimations in the general population. It also reviews various possible solutions to overcome these barriers. The report is based on discussion among experts in the area at a special CardioVascular Clinical Trialists workshop organized by the European Society of Cardiology Working Group on Cardiovascular Pharmacology and Drug Therapy that took place in September 2009. It includes a review of the evidence in favour of the ‘treat-to-target’ paradigm, as well as potential difficulties with this approach, including the multiple pathological processes present in high-risk patients that may not be adequately addressed by this strategy. The risk-guided therapy approach requires careful definitions of cardiovascular risk and consideration of clinical endpoints as well as the differences between trial and ‘real-world’ populations. Cost-effectiveness presents another issue in scenarios of finite healthcare resources, as does the difficulty of documenting guideline uptake and effectiveness in the primary care setting, where early modification of risk factors may be more beneficial than later attempts to manage established disease. The key to guideline implementation is to improve the quality of risk assessment and demonstrate the association between risk factors, intervention, and reduced event rates. In the future, this may be made possible by means of automated data entry and various other measures. In conclusion, opportunities exist to increase guideline implementation in the primary care setting, with potential benefits for both the general population and healthcare resources.

## Introduction

This report is based on the results of discussions that took place among international experts in the field during a special CardioVascular Clinical Trialists workshop organized by the European Society of Cardiology Working Group on Cardiovascular Pharmacology and Drug Therapy in September 2009. The manuscript has subsequently been reviewed and updated by all authors. A separate paper from this Workshop presented a review of current methods of risk stratification for the prevention of cardiovascular disease (CVD), together with a summary of emerging biomarkers and imaging techniques, and the relative merits and limitations of each.

Management of CVD risk factors remains suboptimal in clinical practice with many patients failing to achieve recommended treatment targets. How best to improve the implementation and adherence to guidelines is an important consideration. In this report, we present a discussion of potential opportunities for, and obstacles to, the implementation of more individualized risk stratification to allow more appropriate management strategies and improved outcomes.

The latest international guidelines for the prevention of CVD have already made progress by integrating total risk assessment for individual patients in therapeutic decision making^1–8^ and allowing intensification of the preventive strategy on the basis of risk scores.^9^ European guidelines for the secondary prevention of CVD by means of cardiac rehabilitation have also been published recently.^10^ Nevertheless, there remain a number of issues that may prevent the practical use of risk-guided therapy that may otherwise allow us to optimize the benefit-to-risk ratio in a population with no clinical evidence of CVD.

## The ‘treat-to-target’ paradigm

There is good evidence to support targets for low-density lipoprotein cholesterol (LDL-C) lowering as defined in both European and non-European guidelines, particularly for high-risk patients; in general, ‘the lower the better’ is now accepted for LDL-C.^3–5,8^ The management of dyslipidaemia has substantially improved in recent years, driven by the widespread use of statins, but a significant number of patients on lipid-lowering therapy still do not achieve the targets set in the guidelines.^11,12^ Furthermore, the data show that, despite treatment with statins, a significant residual risk of CVD persists in 65−75% of patients. A recent meta-analysis of subjects assessed with intravascular ultrasound and treated with statins to LDL-C levels ≤1.81 mmol/l (≤70 mg/dl) showed that >20% of subjects continued to show evidence of plaque progression.^13^ This may be due to the fact that the atherogenic dyslipidaemia typically encountered in high-risk patients with metabolic disorders, such as diabetes, metabolic syndrome, and/or obesity, is often characterized by elevated triglycerides and low high-density lipoprotein cholesterol (HDL-C), which may not be efficaciously treated with statins alone.^14^ So far, however, there are no evidence-based targets for triglycerides and HDL-C, although recently the European Atherosclerosis Society (EAS) Consensus Panel suggested that therapeutic targeting of elevated triglycerides (≥2.2 mmol/l) and/or low HDL-C (<1.0 mmol/l) may provide significant further benefit.^15^

There is also good evidence that reducing blood pressure (BP) reduces the incidence of cardiovascular morbid or fatal events, and several trials have demonstrated a fixed assessment of benefit for a fixed reduction in BP.^16^ Based on this evidence, scientific societies in Europe and the USA have recommended higher BP targets in the general hypertensive population, and lower ones for patients at high cardiovascular risk, including those with established coronary disease.^1,17^ The evidence on which these target values is based has recently been questioned.^2,18,19^ In addition, and most importantly in both the hypertensive population and coronary hypertensive patients, BP control is only rarely achieved.^11,20,21^ There are several possible explanations for this poor control of BP: (1) treatment is initiated at a low dosage and is often not titrated up; (2) monotherapy remains the preferred treatment by most physicians while in the majority of hypertensive patients treatment with multiple drugs is required;^22^ (3) adherence to treatment is affected by side effects and many other factors;^23^ and (4) many patients remain overweight or obese and continue on a high-salt diet.^24^ The method used to measure BP may also influence the number of patients found to be ‘at goal’. For example, although antihypertensive treatment has less effect on ambulatory than on office BP,^25^ ambulatory BP monitoring is more likely than office measurement to identify patients at goal, presumably because 30⊟40% of hypertensive patients may have white-coat hypertension and thus a normal ambulatory blood pressure from the start.^26^ Patients may also vary in their ability to tolerate low BP, depending on the extent of end-organ disease present.^27^

Following the UK Prospective Diabetes Study (UKPDS),^28^ there has been an emphasis on tight control of glycaemia. Although this undoubtedly reduces the risk of microvascular complications, evidence that tight glycaemic control reduces macrovascular outcomes is controversial and is based mainly on the results of open-label studies and/or studies that had other methodological limitations. In the Action to Control Cardiovascular Risk in Diabetes (ACCORD) study, tight glycaemic control actually increased mortality in patients with type 2 diabetes.^29–31^ The benefits of tight glycaemic control may be influenced by the mechanism used to achieve it.^32^ For example, aggressive pharmacotherapy may lead to an increased incidence of hypoglycaemic episodes, and the associated sympathetic activation may in turn increase the risk of CVD events. Differences in biology may explain why there is a linear relationship for lowering LDL-C and decreasing CVD risk, but not for lowering BP and haemoglobin A1c.

When considering the possibility of risk-guided therapy, there is no overall contradiction between this and the treat-to-target approach ⊟ the two strategies are complementary. Furthermore, statin therapy may be appropriate even in those with optimal/near optimal cholesterol levels (<130 mg/dl [∼3.3 mmol/l]) if they have a high cardiovascular risk (i.e. >20% risk of events at 10 years) based on the Framingham risk score,^33^ in which case current lipid goals are inapplicable. It is in individuals at moderate total cardiovascular risk that novel biomarkers and imaging techniques may prove valuable in order to reclassify them into either high or low categories.

In summary, targets are needed to guide physicians, particularly general practitioners (GPs), in the appropriate management of patients.^34^ However, targets should be specific for the strategy used to identify the goal (e.g. statins and LDL-C), and targets have not yet been defined for all risk factors.

## Using risk-guided therapy in clinical practice: approvability issues

Prevention of CVD represents one of the most important aspects of preventive medicine today. In order to achieve the best prevention, adequate risk stratification has to be performed followed by the most appropriate intervention, according to available clinical guidelines. The European Medicines Agency (EMEA) has created a guidance for the evaluation of drugs in the prevention of cardiovascular events^9^ with a view to obtaining the best evidence to contribute to subsequent clinical guidelines. Three key issues are required by the EMEA from a regulatory perspective: (1) accurate definition of the cardiovascular risk of the target population; (2) accurate definition of clinical endpoints and duration of follow-up; and (3) an accurate evaluation of safety. Ideally, there should also be a placebo-controlled study to demonstrate the superiority of a new drug to be added on top of optimal standard treatment, and total mortality is considered a better endpoint than cardiovascular mortality.

In certain situations such as heart failure and following myocardial infarction, where total mortality/year is high, the rules of the EMEA can easily be followed. In other situations, however, such as studies assessing the effect of drugs on cardiovascular outcomes in arterial hypertension, the primary endpoint is a composite of fatal and nonfatal cardiovascular events, which can complicate interpretation of the results. Recently, other types of study with similar composite primary endpoints have been considered that were designed to test the effect of suppression of the renin‐angiotensin system on cardiovascular outcome in patients with high cardiovascular risk. These studies include, for example, the Heart Outcomes Prevention Evaluation trial (HOPE),^35^ the EURopean trial On reduction of cardiac events with Perindopril in stable coronary Artery disease (EUROPA),^36^ the Prevention of Events with ACE inhibition (PEACE) trial,^37^ the ONgoing Telmisartan Alone and in combination with Ramipril Global Endpoint Trial (ONTARGET),^38^ and the Telmisartan Randomized AssessmeNt Study in aCE-iNtolerant subjects with cardiovascular Disease (TRANSCEND).^39^ The conclusions are that, when compared with placebo, an angiotensin-converting enzyme inhibitor (ACEi) can be beneficial,^35,36^ neutral,^37,39^ and similar to an angiotensin receptor blocker (ARB), while the combination of an ACEi and an ARB does not add any further benefit.^38^

These partly conflicting results are difficult to interpret and necessitate new considerations in the performance and interpretation of future trials. An example of this is the need to estimate real BP levels in future studies that contemplate hard endpoints.^40^ In fact, casual BP measurement does not reflect real values and hypotension may be seen to occur more frequently based on other forms of BP measurement.^41^ Similarly, if we are to protect our patients, we need to know what is more relevant: to attain the lowest BP or to attain the lowest cardiovascular risk.^42^ In high-risk patients, there is a ceiling effect for treatment benefits, probably as a result of the need to manage multiple risk factors, one or more of which have already reached a level where the benefit of intervention is blunted.^42^ In fact, the recently published reappraisal of the European Society of Hypertension guidelines^2^ recognizes the need for new trials, as summarized in Table 1.^43^

**Table 1.** New trials needed

1	Trials in grade 1 hypertensive patients with low risk
2	Trials in elderly hypertensive with systolic blood pressure between 140 and 160 mmHg (is <140/90 mmHg an adequate goal?)
3	Trials in type 2 diabetic patients with high normal blood pressure (is <130/80 mmHg an adequate goal?)
4	Trials with lifestyle changes (do they decrease morbidity and mortality?)

Modified from Wilhelmsen et al.^43^

The utilization of biomarkers as surrogate or intermediate endpoints in future trials may contribute to the solution of these problems. However, this assumes that positive changes in these parameters are associated with less progression of atherosclerosis and with a reduction in fatal and nonfatal cardiovascular and renal events. While there has been interest in the association between C-reactive protein (CRP) and cardiovascular events, and the possible impact of statin therapy on CRP levels and patient outcomes,^44^ current European guidelines do not support the use of CRP levels as a basis for therapeutic decisions but focus instead on risk scoring. The reasons for this are: (1) that the Justification for the Use of Statins in Prevention: an Intervention Trial Evaluating Rosuvastatin (JUPITER) trial did not include a low CRP group to compare with high CRP subjects; and (2) that recent studies based on Mendelian randomization do not support the hypothesis that CRP is causally related to atherosclerotic CVD.

Last, but not least, clinical trials exclude a relevant percentage of patients that we have to treat in our daily clinical practice. This is due to the use of exclusion criteria that are frequently present in ‘real-world’ patients. In such patients, our intuition remains an important tool to obtain the greatest benefit through the administration of multiple combined pharmacological therapies.

## Implementation and economic challenges and barriers to risk-guided therapy

Despite the existence of well-established and safe pharmacological therapy for controlling cardiovascular risk factors and preventing CVD, surveys have revealed inadequate management of patients with or at risk of CVD in most European countries.^11^ Guideline implementation may be improved by addressing some of the issues that physicians perceive as constraints to treating their patients appropriately. These include lack of time, prescription costs, poor patient compliance, too many guidelines, inconsistencies among international and national guidelines, poor awareness of guidelines, and lack of motivation.^45,46^ Practical answers to these perceptions must be given.

## Cost-effectiveness issues

Limits on healthcare resources mandate that resource-allocation decisions be guided by considerations of cost in relation to expected benefits.^47^ In cost-effectiveness analysis, the ratio of net healthcare costs to net health benefits (including life expectancy-adjusted quality-of-life indicators, both adverse and beneficial effects of therapy) provides an index by which priorities may be set. Reducing tobacco use and screening and treatment for hypertension and elevated cholesterol are among the most cost-effective strategies for disease prevention. Over time, ensuring adherence with therapy is also cost-effective, as it reduces the costs that would otherwise result from treatment of cardiovascular events.

It is important to balance the efficacy and cost of any intervention against the level of cardiovascular risk in the target population and the reduction in events achieved with the intervention. Non-personal interventions, such as mass-media messages to change diet or legislation to lower the salt content of processed foods, are shown to be cost-effective ways to limit CVD and could avert large disability-adjusted life years per year worldwide.^48^ Combination treatment (cholesterol and BP lowering) for people whose risk of a cardiovascular event over the next 10 years is above 35% is also cost-effective, leading to substantial additional health benefits.^48^ Overall, World Health Organization estimations suggest that this combination of personal and non-personal health interventions could lower the global incidence of cardiovascular events by as much as 50%.^48^

Research addressing pertinent questions and using appropriate analytical methods is necessary to assess the cost-effectiveness of current and future strategies of prevention. Furthermore, cost-effectiveness analyses of interventions directed towards individuals, the healthcare system and community programmes are necessary, as the information provided by each analysis differs greatly. Nevertheless, there is compelling evidence that treating high-risk subjects with efficient preventive measures is cost-effective.^49^

## Challenges to clinical guideline implementation

The challenge for guidelines does not cease with a consensus document or repeating cycles of review. Practical implementation is the critical step in establishing higher standards of care for individual patients. Cost-effectiveness is a key not only to the content of guidelines but also in the assessment of implementation.

Improved guideline uptake is not only an index of better standards but a validation of the process of guideline production. Unfortunately, surveys confirm that cardiovascular guideline implementation is lacking and that guideline revision does not improve uptake. The EUROASPIRE surveys^11^ reveal classical risk factors remaining undocumented, poorly documented, or not integrated in care against a background of static or increasing risk factor prevalence. Paper guidelines distribution, web pages, educational meetings, and reviews with important backing from major industry partners seem ineffectual, with the population prevalence of risk factors going in the wrong direction. Improving consensus between guidelines is also important; differences in recommendations may act as a barrier to guideline adherence, although some reflect true differences in the target patient populations. Areas of disagreement between guidelines often arise because of gaps in the evidence on which the recommendations are based; in such cases, additional studies are required to better define the appropriate treatment options.

## Whose responsibility is guideline implementation?

**Figure 1.**
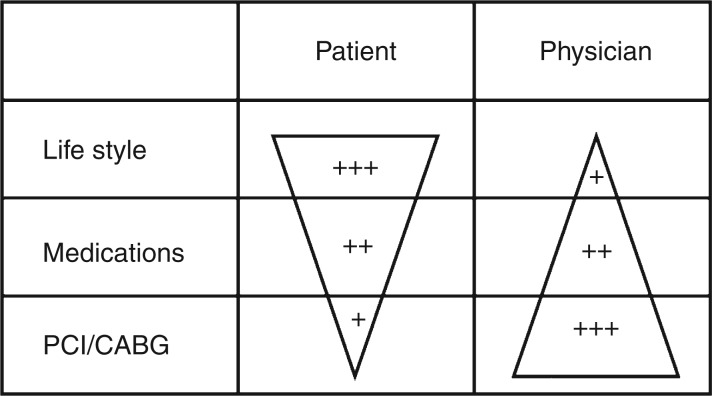
Patient‐practitioner interactions. Patient⊟practitioner interactions can shed light on reasons for inaction in the management of chronic ischaemic heart disease. CABG, coronary artery bypass graft; PCI, percutaneous coronary intervention.

Implementation of clinical guidelines is defined by individual patient⊟practitioner interactions (Figure 1). Some aspects of ancillary risk behaviour (e.g. tobacco smoking) can be controlled at population level by legislation,^50^ taxation, and the restriction of advertising. While governments can support guideline development and dissemination and public health campaigns, and most European countries have national dietary guidelines, they cannot dictate individualized behaviours such as total calorie intake, dietary composition, salt addition (within reason), physical activity, or any of a range of associated risk behaviours. Explanations for inaction on agreed responses lie within the patient⊟practitioner interaction. That is, although most physicians support the use of guidelines, according to recent data, only half actually use them in their everyday work, and their level of knowledge of target goals is less than satisfactory.^51^ Furthermore, while 80% of physicians believe they are treating their dyslipidaemic patients well, over 50% of the population in a European study claimed never to have discussed any risk factors with their physician.^52^ Therefore, changing physicians’ awareness and behaviour should be one of the major strategies to achieve better implementation of treatment guidelines.

Addressing the failure of implementation is about changing strategies to support uptake among the staff that contact individuals. These must address individualized risk assessment, in a way that is more relevant to the individual than to populations. Collated scoring and repeated measures of these are essential for reinforcing individualized goals, as well as identifying failure to set individual targets. Targets must be simple, realistic, adjusted for absolute risk, and applicable in primary prevention. Auditing guideline implementation must employ automated numerate data acquisition, accuracy and verification, and easy collation of the summary risk scores to guide management.

The patient's perspective on the acceptability of strategies for CVD prevention should also be considered, both in terms of the treatments and guidance they are offered and the targets they are set. At present, there is little in the way of patient-reported outcomes or perspectives in the context of CVD. This could be improved in practical terms by acquiring data using standardized patient questionnaires (assessing their symptoms, satisfaction with care, etc.), and linking the information with electronic health records and clinical research.^53^ On a more personal level, the use of motivational interviewing techniques during consultations can enhance physicians’ understanding of their patients’ views and aspirations, as well as encouraging patients to change their behaviours and improving the overall physician⊟patient relationship.^54^ The ongoing GULiVer (Gent, Utrecht, Liverpool, and Verona) project should also provide some important insights into patients’ views on doctors’ communication skills.^55^

## Quality, application, and implementation are most needed in primary prevention

Guideline implementation is easier in secondary prevention, where vascular events underline the need for individual changes in behaviours and treatments. Changing behaviours and implementing guidelines in community care, where asymptomatic disease will likely be present, is also important. Determinants of behaviour here are more complex and are driven by less overt illness and the mediators of change enacted. Implementation is dependent on the quality of risk factor definition and an integrated risk factor score.

## Is current implementation working for individuals or populations?

EUROASPIRE samples suggest that guidelines for cardiovascular prevention are only sporadically applied and ineffectual in patients with established coronary heart disease. Recent evidence from the population surveys also demonstrated underachievement of guideline targets in a substantial proportion of patients with stable atherothrombotic disease.^56^ These data do not necessarily indicate that individualized assessments are not being completed, only that the process is not being documented.

The target for preventative guidelines is to identify and define the earliest stage at which there is an appreciable increased relative risk of future vascular events in the primary care setting. Few individuals in young adult life or middle age will respond positively to absolute risk assessments that may incorrectly portray their relative risk, even though this is a time when there is maximized potential for modifying the evolution of vascular disease. That is, these individuals have less disease but are more easily modified compared with those with end-stage disease, where interventions are less likely to affect vascular deterioration. Guideline implementation should focus on community care practitioners, whether physicians, nurses, or pharmacy based. In particular, primary prevention based on guidelines, which is unsatisfactory at present, requires a systematic, comprehensive, multidisciplinary approach, which addresses lifestyle and risk factor management, with medicines prescribed when necessary.^57^ Current paradigms of dissemination by educational material, industry distribution of guidelines, and local or regional meetings have a generally ill-defined impact. Successful examples, such as initiatives by the American Heart Association for secondary care in overt coronary disease (the Get With The Guidelines, GWTG, programme); women's cardiovascular health initiative (the Wear Red; Red Dress campaigns),^58^ and heart failure care (IMPROVEMENT programme)^59^ are limited to secondary prevention. This is a smaller target than primary prevention, with potentially less gain due to the reduced impact of interventions on more advanced disease. They are often based in centres with already high standards of practice but dealing with a minority of potential patients. Testing a GWTG type of programme, adapted to the European context in primary prevention and in primary care, may be worth pursuing. Because this approach is based on automated information management, widespread use of digital medical records could be encouraged.

## Alternative methods to improve guidelines implementation?

Alternatives to traditional models must be considered to improve guideline uptake and action. These can and have been based on utilizing reimbursement to affect activity.^60,61^ These appear as incentives in US insurance-based healthcare and similar principles have been piloted in the UK state-funded system. The key is to define change and the quality of data collection for individual subjects, both the practitioners and the people subject to the guideline, and to link this to impact on event rates. Cost-effectiveness data are effectively built into the process of monitoring implementation. In the UK, this is centred on the national digital medical records project,^62,63^ where hospitalization events are verifiable.

In the UK, digital medical records have been used to promote data entry by linkage to reimbursement (known as quality payments or the Quality and Outcomes Framework, QOF). This process results in improved records of care.^64^ While there may still be issues as to the quality and scope of records obtained,^65^ there is no reason to suppose that the QOF principle cannot be extended to digital documentation of an individualized cardiovascular risk factor score. These can be linked to the target goals of treatment strategies based on either blood biomarker or physical measurements (e.g. BP). Disappointingly, a recent study found that the introduction of the QOF pay-for-performance incentive did not result in any appreciable improvements in processes of care or outcomes for patients with hypertension.^66^ Nevertheless, extending the principle, cycles of repeat examination and reinforcement can be engineered by automated reminders and individualized trends, to help in consultation and hopefully improve outcomes in the longer term.

These systems need not employ medically qualified practitioners. The role of nurses and pharmacists in hypertension care is well established.^67^ BP readings can, of course, be directly entered into the digital case record without practitioners’ input of primary data. Qualified personnel are critical to interpret and communicate with individual patients. They must participate in data validation, and audit and external audit to the guideline standard is intrinsic to this process. A collated risk score for individual monitoring and treatment boundaries is preferred. Localized population record linkage allows screening of the thresholds for intervention and allows these to be reset in response to data on effective treatments. Practitioners working outside the limits can be sampled for the explanations behind variance.

It is feasible to facilitate clinical trial structures including such patients, potentially making the regulatory process a more integral part of development. This could improve understanding at a regulatory level of the role of a new medical product in practice. Equally, post-marketing surveillance for both safety and efficacy is feasible in real time with electronic collation of a drug treatment (using encashment rather than prescription data).

The collation of individualized data at point of entry, with fixed time, date, and repeated measures linked to events, has been demonstrated for diabetes care in the Diabetes Audit and Research in Tayside Scotland (DARTS) study.^68,69^ Anonymized data extraction can be utilized to assess compliance and facilitate external quality audit, including event linkage.

## Barriers to digital implementation programmes

Global economic pressures mean that costs of implementation are ever more critical to change. Moreover, it is important not to waste resources on activity that does not effect change shown to be beneficial for the health of individuals. The infrastructural costs of these programmes are massive and will not be possible in every health economy. Current costs of the UK digital case record linkage project are estimated at £10 billion, and the project is currently without a completion date and records are not yet linked to encashment of prescribed medicines. Nonpharmacological aspects of ‘therapy’, potentially a key factor in cardiovascular risk reduction, are poorly defined. However, data such as weight or BP can be supplemented by simple tests such as urine microalbuminuria or blood biomarkers, and all such data are subject to the increased power of repeated measures within subjects.^70^

## Future technologies

Automation of data entry and collection is realistic. Longer term, this may include patient-mediated data entry and feedback. Data linkage may be validated to digital identifiers carried in the form of a national identity card or even subcutaneously implanted. Limited implantable biomonitoring is a routine aspect of device care in advanced CVD. Similar applications for cardiovascular risk parameters are easily envisioned. All of these frontiers need to be explored at a societal level as they involve the rights of the patient to consent and opt into their own healthcare monitoring. For now, we need to address the poor implementation of the associations we currently understand between risk and intervention, and adopt new ways to implement established guidelines for individual patients and populations. In this respect, ‘telehealthcare’ via the internet and smart phones represents an increasingly important channel of contact and follow-up for the management of long-term conditions.^71^ One example is PREDICT-CVD, a web-based decision support system for primary care patients in New Zealand.^72^ One month after installation of the system, CVD risk assessment was found to have increased four-fold compared with the same 4-week period a year earlier.

## Conclusions

Strategies to improve guideline adherence could include producing user-friendly guidelines, better communication of updates and multidisciplinary CVD prevention programmes. Linking GPs’ remuneration to goal achievement may also prove to have a positive influence. An initiative by the European Association for Cardiovascular Prevention and Rehabilitation aims to improve the translation of guideline recommendations into effective care by improving communication and collaboration between opinion leaders, professional societies, and national and European coordinators for CVD prevention. The stumbling blocks that limit uptake and monitoring of treatment guidelines include lack of time and, sometimes, lack of interest on the part of the primary care physicians. However, several tools are available to help increase their implementation. These include incentives (e.g. QOF in the UK, and the GWTG programme in the USA), automated computer-based assistance, online feedback processes (e.g. so that physicians can review their own progress and compare it with others), and ensuring help from nursing staff.

Earlier and/or more appropriate intervention for the management of CVD risk may result in improved long-term outcomes and overall benefits for both the general population and healthcare resources.

## Declaration

Employees of AstraZeneca were permitted to read the manuscript at a late stage in its development. Responsibility for opinions, conclusions, and interpretation of the data lies with the authors.
